# Healthcare burden of treated gastroesophageal reflux disease among U.S. adults, 2018–2023: a pooled cross-sectional analysis of the medical expenditure panel survey

**DOI:** 10.3389/fmed.2026.1933050

**Published:** 2026-09-04

**Authors:** Mureaihemaitijiang Mutalifu, Wubulikasimu Mijiti

**Affiliations:** 1 Department of Gastroenterology, The First Affiliated Hospital of Xinjiang Medical University, Urumqi, Xinjiang Uygur Autonomous Region, China; 2 Department of Hepatic Laparoscopic Surgery, The First Affiliated Hospital of Xinjiang Medical University, Urumqi, Xinjiang Uygur Autonomous Region, China

**Keywords:** gastroesophageal reflux disease, healthcare expenditure, healthcare utilization, medical expenditure panel survey, outcome-dependent ascertainment, patient-reported outcomes, psychological distress, treated condition record

## Abstract

**Background:**

U.S. evidence combining healthcare expenditure, utilization and patient-reported burden in gastroesophageal reflux disease (GERD) within one nationally representative framework is limited. Because survey condition records capture only medically attended disease, we quantified the burden of a treated, event-linked reflux condition record.

**Methods:**

We pooled the 2018–2023 Medical Expenditure Panel Survey Household Component. Since 2018 a condition enters the MEPS Medical Conditions File only when linked to a medical event or prescribed-medicine purchase, so the exposure is a treated (event-linked) ICD-10-CM K21 record, not symptom-defined GERD. Pooled variance used the AHRQ HC-036 file, with person weights divided by six. Primary outcomes were annual all-cause total expenditure (2023 USD) and high-cost status (top weighted decile); secondary outcomes were out-of-pocket expenditure, utilization and patient-reported burden. Expenditure was modelled by survey-weighted quasi-Poisson (adjusted mean ratio) and linear regression (adjusted mean difference); *P* values were Benjamini–Hochberg corrected.

**Results:**

The sample comprised 117,962 person-years (62,494 persons), including 8,725 (5,292 persons) with a treated K21 record. The weighted proportion carrying such a record was stable at 6.4%–6.7%, far below symptom-based U.S. prevalence estimates of about 20%–30%. Fully adjusted models gave an adjusted mean ratio for total expenditure of 1.39 (95% CI: 1.32–1.46), a mean difference of $5,481 ($4,596–$6,367), and associations with out-of-pocket expenditure ($388; $233–$542), high-cost status (odds ratio [OR] 1.70; 1.58–1.84), emergency department use (OR: 1.54; 1.43–1.66), inpatient use (OR: 1.38; 1.27–1.50), office-based (OR: 3.58; 3.00–4.27) and outpatient visits (OR: 1.81; 1.68–1.94), all FDR-corrected *P* < 0.0001. Prescription use is reported descriptively only, as a filled prescription can itself generate the record. Patient-reported burden was consistently worse. Adults with both a treated record and depressive symptoms had the highest burden (expenditure difference $11,274, $8,059–$14,489; high-cost OR: 2.70, 2.15–3.39), with no significant interaction.

**Conclusions:**

Adults with a treated, event-linked reflux record incur substantially higher all-cause expenditure and utilization and report worse physical, psychological and functional health. Because identification requires healthcare contact, these estimates describe the burden borne by adults receiving care for reflux disease, not the causal effect of GERD.

## Introduction

Gastroesophageal reflux disease (GERD) is commonly defined as a condition in which reflux of gastric contents causes troublesome symptoms and/or complications (1). Heartburn and regurgitation remain the typical symptoms, but contemporary clinical guidelines emphasize that GERD is a heterogeneous chronic disorder that may require long-term symptom-directed treatment, diagnostic evaluation in selected patients, and management of extra-esophageal or complicated disease (2). Recent reviews similarly describe GERD as a phenotypically diverse disorder in which symptom burden, objective reflux evidence and treatment response are only loosely coupled (3).

GERD represents a substantial population health burden. Global meta-analyses have shown that reflux symptoms are common among adults, with marked geographic variation and generally higher prevalence estimates in Western populations (4, 5). In North America, symptom-based estimates of GERD prevalence range from about 18% to 28% (6), and a contemporary U.S. population survey reported that more than 30% of adults experience reflux symptoms at least weekly (7). Because symptoms often recur and may persist over time, GERD can generate continuing demand for physician visits, diagnostic testing, acid-suppressive therapy, and, in selected cases, procedural or surgical management (2).

Population estimates of symptom-based GERD and estimates derived from healthcare records therefore describe different, although overlapping, populations. Many adults with reflux symptoms manage them with over-the-counter medication and never receive a coded diagnosis, whereas condition records in claims and in national surveys are generated only when a person makes contact with the healthcare system. This distinction is explicit in the Medical Expenditure Panel Survey (MEPS): from 2018 onwards, a condition is retained in the MEPS Medical Conditions File only if it is linked to a medical event or a prescribed-medicine purchase during the calendar year (8). Conditions recorded in these files are consequently medically attended, and the proportion of adults carrying such a record is a measure of treated disease rather than of community prevalence. This treated population, however, is precisely the group that generates healthcare utilization and spending, and characterizing its burden is directly relevant to health-services planning.

Beyond symptom control, GERD is clinically relevant because it affects patient-centered outcomes. Prior studies have linked GERD with impaired health-related quality of life, sleep disturbance, reduced daytime functioning, and economic burden (9–11). Psychological symptoms are also frequently reported among patients with reflux disease, and recent evidence suggests that anxiety and depression are associated with GERD severity and symptom burden (12, 13). However, most existing studies have focused on selected domains, such as prevalence, medication use, cost, quality of life, or psychological symptoms, rather than evaluating these dimensions together in a contemporary nationally representative U.S. population.

Using pooled 2018–2023 MEPS data, we therefore estimated the multidimensional burden associated with a treated, event-linked K21 reflux condition record among U.S. adults. The prespecified primary outcomes were annual all-cause total healthcare expenditure and high-cost healthcare status, defined as expenditure at or above the weighted 90th percentile. Prespecified secondary outcomes were annual out-of-pocket expenditure; emergency department, inpatient, office-based and outpatient utilization; patient-reported physical, psychological, pain, energy and functional burden; and the joint burden of a treated GERD record together with depressive symptoms. The target estimand throughout is the total association between having a treated GERD record and each outcome, conditional on antecedent characteristics and comorbidity, and not the causal effect of reflux disease itself; the reasons for this distinction are developed in the Methods and revisited in the Discussion.

## Methods

### Study design and data source

We conducted a pooled repeated cross-sectional analysis of the 2018–2023 Medical Expenditure Panel Survey Household Component (MEPS-HC). MEPS is sponsored by the Agency for Healthcare Research and Quality (AHRQ) and collects nationally representative information on demographic characteristics, health conditions, health status, healthcare utilization, healthcare expenditures, insurance coverage, income, and employment among the U.S. civilian noninstitutionalized population (8, 14, 15). For each survey year, we used the Full-Year Consolidated File and the Medical Conditions File. For pooled variance estimation we additionally used the AHRQ MEPS HC-036 1996–2023 Pooled Linkage Variance Estimation File (16). Reporting followed the Strengthening the Reporting of Observational Studies in Epidemiology (STROBE) framework where applicable (17).

### Study population

The analytic sample included adults aged 18 years or older who had positive person-level survey weights and valid strata and primary sampling unit information. Respondents younger than 18 years and those without valid survey design information were excluded. Because each MEPS panel spans two calendar years, an individual may appear in two consecutive Full-Year Consolidated Files and therefore contribute up to two person-year observations to the pooled file. We accordingly report both the number of person-year observations and the number of unique persons, identified by the combination of person and panel identifiers, throughout the manuscript.

### Definition of treated GERD

The primary exposure was a treated (event-linked) reflux condition record, defined by the presence of ICD-10-CM code K21 in the MEPS Medical Conditions File. Two features of these files determine how this exposure should be interpreted. First, MEPS public-use Medical Conditions Files release only three-character ICD-10-CM codes, so K21.0 (gastro-oesophageal reflux disease with oesophagitis) and K21.9 (gastro-oesophageal reflux disease without oesophagitis) cannot be distinguished; K21 here denotes any coded reflux disease record, and no severity, phenotype or endoscopic information is available. Second, since 2018 a condition is retained in the Medical Conditions File only when it is linked to a medical event or a prescribed-medicine purchase in the same calendar year (14). A K21 record therefore indicates medically attended reflux disease during the survey year and not a clinically adjudicated diagnosis based on endoscopy, ambulatory reflux monitoring or standardized symptom criteria of the kind used in contemporary diagnostic frameworks (18). Throughout this manuscript we use the terms “treated GERD record” or “recorded GERD” for this exposure, and we report the weighted proportion of adults carrying such a record rather than a prevalence of GERD.

### Outcomes

Two outcomes were prespecified as primary: annual all-cause total healthcare expenditure and high-cost healthcare status. Expenditures were converted to 2023 U.S. dollars using annual Consumer Price Index for All Urban Consumers values from the U.S. Bureau of Labor Statistics (19). High-cost healthcare status was defined as annual total expenditure at or above the pooled survey-weighted 90th percentile, which corresponded to $20,773 in 2023 dollars; year-specific weighted 90th percentiles are reported in Supplementary Table S3 and were used in a sensitivity analysis.

Secondary outcomes comprised annual out-of-pocket expenditure, defined as the sum of the person's own payments across all medical events and prescribed medicines during the year and expressed in 2023 dollars; any emergency department use, any inpatient stay, any office-based visit and any outpatient visit during the survey year; and the patient-reported measures described below. Any prescription medicine purchase was examined as a descriptive outcome only and is not treated as an inferential outcome, for the reasons given under Statistical analysis.

Patient-reported burden outcomes were defined individually as follows. Fair or poor perceived physical health and fair or poor perceived mental health were each derived from the corresponding five-point item (1 = excellent to 5 = poor) collected in every survey year, with responses of 4 or 5 classified as fair or poor. Activity limitation was defined as any reported limitation in work, housework or school, collected in every survey year. Serious psychological distress was defined as a Kessler Psychological Distress Scale (K6) score of 13 or higher; the K6 ranges from 0 to 24, with higher scores indicating greater distress (20). Depressive symptoms were defined as a Patient Health Questionnaire-2 (PHQ-2) score of 3 or higher; the PHQ-2 ranges from 0 to 6, with higher scores indicating more frequent depressive symptoms (21). Moderate or greater pain interference was defined from the Veterans RAND 12-Item Health Survey (VR-12) pain item as a response of moderate or worse, and low energy from the corresponding VR-12 energy item (22). The K6, PHQ-2 and VR-12 items are administered through the Self-Administered Questionnaire and the Preventive Self-Administered Questionnaire and are therefore available only for the subset of respondents who completed those instruments, which is the main reason that analytic sample sizes differ across outcomes. Continuous versions of the K6 score, PHQ-2 score, pain interference score, energy score and social limitation score were analysed as secondary outcomes; the VR-12 physical and mental component summary scores were not available in the pooled public-use files for these years and were therefore not analysed.

### Covariates and target estimand

We specified an explicit conceptual model before analysis in order to separate confounders from variables lying on the pathway between a treated GERD record and the outcomes. Variables treated as antecedent confounders were age, sex, survey year, race/ethnicity, poverty category, insurance coverage, U.S. census region and comorbid conditions (diabetes, hypertension, heart disease, stroke, asthma, arthritis and cancer). Variables treated as potential mediators, and therefore excluded from the main models, were perceived physical and mental health and the patient-reported measures themselves. Obesity is an established risk factor for reflux disease and is therefore a confounder rather than a mediator; body mass index is derived from the Self-Administered Questionnaire and is missing for most respondents, so we could not include it in the main models and instead report a prespecified sensitivity analysis additionally adjusted for body mass index within the subsample in which it was recorded.

Models were fitted in stages so that the contribution of each covariate block is visible. Model 1 adjusted for age, sex and survey year. Model 2 additionally adjusted for race/ethnicity, poverty category, insurance coverage and region. The fully adjusted model additionally included comorbid conditions. Because perceived physical and mental health plausibly lie on the causal pathway between a treated GERD record and the outcomes, they are excluded from the main models and instead reported as a sensitivity analysis. The target estimand is accordingly the total association between a treated GERD record and each outcome, and not a direct effect net of perceived health.

### Joint GERD and mental burden analysis

To evaluate whether coexistence of GERD and psychological burden identified a higher-risk subgroup, we created four joint exposure categories: no GERD/no mental burden, GERD only, mental burden only, and GERD plus mental burden. Mental burden was defined in the primary analysis solely by PHQ-2 depressive symptoms (score ≥ 3), among respondents with a valid PHQ-2 score. A parallel analysis defined mental burden solely by K6 serious psychological distress (score ≥ 13), among respondents with a valid K6 score, and is reported as a sensitivity analysis. The two instruments measure related but distinct constructs, so they are analysed separately rather than combined into a single variable. Joint-exposure models used exactly the same covariate set as the fully adjusted main models, so that joint and main estimates are directly comparable. Interaction was assessed on the multiplicative scale by a Wald test of the product term in the logistic models and on the additive scale for the expenditure difference.

### Statistical analysis

All analyses accounted for the complex MEPS survey design using strata, primary sampling units, and person-level weights. Because the MEPS variance structure changed from 2019 onwards, pooling 2018 with later years requires a common variance structure. We therefore used the pooled variance stratum and primary sampling unit variables supplied in the AHRQ HC-036 1996–2023 Pooled Linkage Variance Estimation File (16), which provide a common variance structure across the 2019 design boundary; every analytic record was matched to the pooled linkage file, with no unmatched observations. Person-level annual weights were divided by six so that estimates represent an average annual quantity over the pooled period. All association estimates reported below come from a single pooled regression model fitted across all person-year observations from 2018 to 2023; they are not year-specific estimates, averages of year-specific estimates, or values from any individual year. Only the proportion of adults with a treated GERD record and the high-cost expenditure threshold are reported year by year.

Survey-weighted means and proportions were used to describe sample characteristics. Expenditure was analysed on its original dollar scale by two complementary survey-weighted generalized linear models: a quasi-Poisson model with a log link, which yields an adjusted ratio of arithmetic mean expenditure and accommodates both the mass at zero and the strong right skew without requiring any handling of log(0) or a smearing retransformation, and an identity-link linear model, which yields an adjusted difference in arithmetic mean expenditure in dollars. Reported dollar differences are therefore adjusted differences in means and not differences in medians. Two sensitivity analyses were prespecified: an ordinary least squares model of log-transformed expenditure restricted to respondents with positive expenditure, whose coefficient represents a relative difference on the geometric-mean scale rather than a percentage difference in arithmetic mean expenditure; and a two-part model combining a logistic model for any expenditure with a log-link generalized linear model for positive expenditure, from which an average marginal difference was computed. Binary outcomes were analysed by survey-weighted logistic regression.

Any prescription medicine purchase was not modelled as an inferential outcome. Because a K21 record enters MEPS only when it links to a medical event or a prescribed-medicine purchase, a filled reflux prescription can itself generate the condition record, so an association between a treated GERD record and prescription medicine use is largely definitional. The corresponding estimate is reported once, in Supplementary Table S2, and is labelled descriptive.

Regression analyses used complete-case records for the variables included in each model. The unweighted analytic sample size for every model is reported in Supplementary Table S6, and the proportion of missing values for each variable by GERD status is reported in Supplementary Table S4. Because multiple outcomes were examined, *P* values across the primary and secondary outcome family were corrected by the Benjamini–Hochberg false-discovery rate procedure (23), and FDR-corrected *P* values are reported alongside nominal values in the tables. Interpretation emphasizes the consistency, magnitude and precision of associations rather than isolated *P* values. Two-sided *P* values below 0.05 were considered statistically significant. Analyses were performed in R using the survey package (24).

## Results

### Analytic sample and proportion of adults with a treated GERD record

The pooled full-year MEPS files included 156,464 person-year records. After excluding respondents younger than 18 years, 121,770 adult person-year records remained, and after further excluding records without positive person-level survey weights or valid survey design information, the analytic sample comprised 117,962 adult person-year observations representing 62,494 unique persons. A treated K21 reflux condition record was identified in 8,725 person-year observations, representing 5,292 unique persons. All analytic records were matched successfully to the HC-036 pooled linkage file.

**Figure 1:**
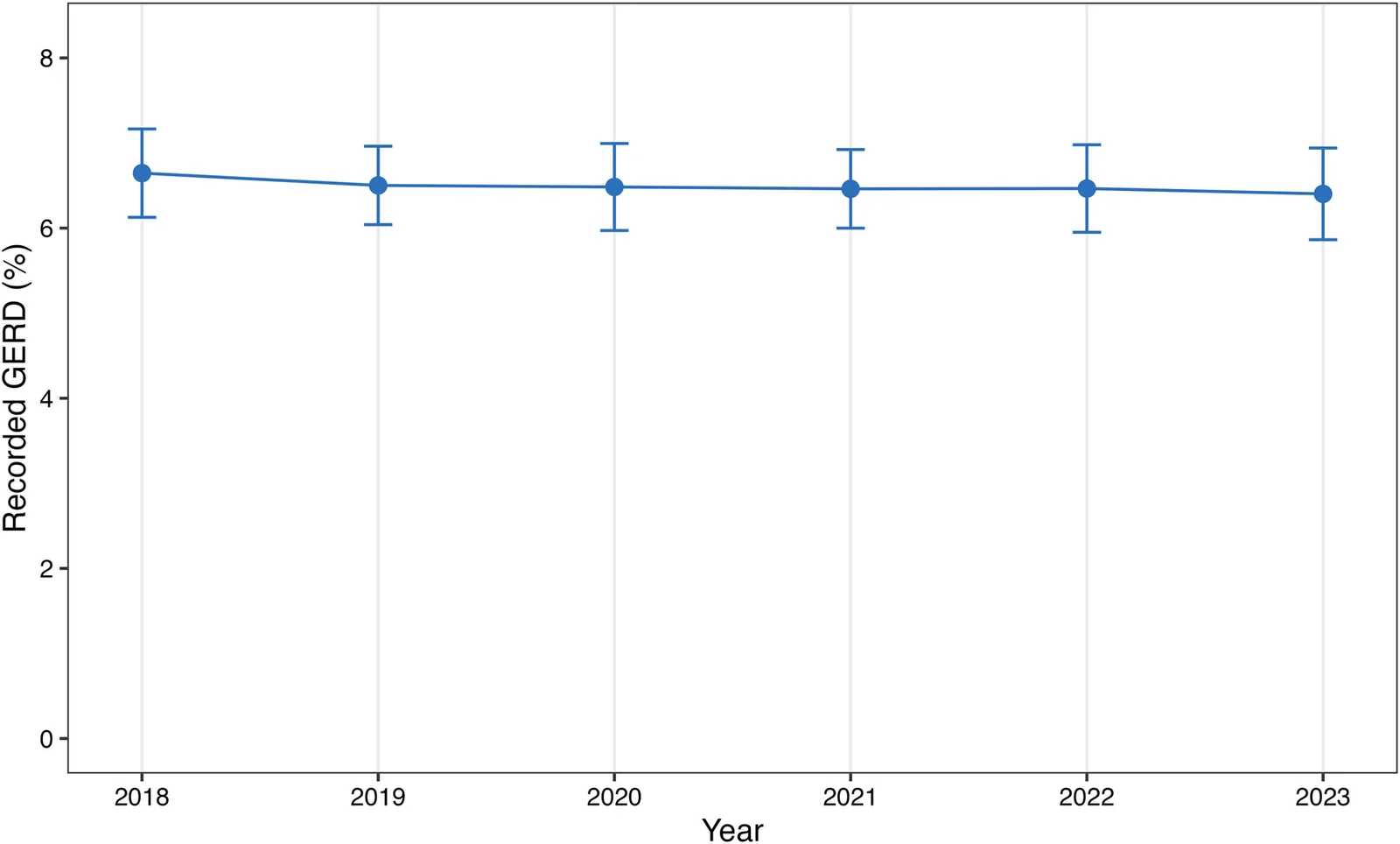
Weighted proportion of U.S. adults with a treated GERD condition record, 2018–2023. Points represent year-specific survey-weighted proportions of U.S. adults with an ICD-10-CM K21 reflux condition record linked to a medical event or prescribed-medicine purchase in that year, and vertical bars represent 95% confidence intervals. This proportion measures medically attended reflux disease and is not an estimate of symptom-based GERD prevalence in the general population. GERD, gastroesophageal reflux disease; ICD-10-CM, international classification of diseases, tenth revision, clinical modification.

The weighted proportion of U.S. adults with a treated K21 record was stable across the study period, at 6.65% (95% CI: 6.13 to 7.17) in 2018 and 6.40% (95% CI: 5.86 to 6.94) in 2023, with a range of 6.4% to 6.7% across all six years (Figure 1 and Supplementary Table S3). This proportion is substantially lower than symptom-based estimates of GERD prevalence in North America, which range from approximately 18% to 28% (6), and than the more than 30% of U.S. adults who report at least weekly reflux symptoms (7). The difference is expected and is discussed below: MEPS condition records capture only medically attended disease, whereas symptom surveys capture the much larger population of adults with reflux symptoms, including those who self-manage with over-the-counter medication or do not seek care. Missingness for each analytic variable by GERD status is summarized in Supplementary Table S4, and the analytic sample size for every model is given in Supplementary Table S6.

### Sample characteristics

Adults with GERD differed from adults without GERD across demographic, insurance, regional, and health-related characteristics (Table 1). The weighted mean age was 61.63 years (SE 0.32) among adults with GERD and 47.13 years (SE 0.16) among those without GERD. Women represented 57.8% of adults with GERD compared with 51.0% of adults without GERD. Adults with GERD were more often non-Hispanic White (77.6% vs. 60.5%), more often publicly insured (38.9% vs. 23.6%), and less often uninsured (1.0% vs. 7.9%). Comorbidities were consistently more prevalent among adults with GERD, including hypertension (62.8% vs. 30.2%), arthritis (57.2% vs. 22.3%), diabetes (23.3% vs. 10.4%), cancer (23.0% vs. 10.3%), asthma (20.9% vs. 13.5%), heart disease (12.9% vs. 4.2%) and stroke (9.9% vs. 3.4%). Within the subsample with recorded body mass index, adults with a treated GERD record had a higher weighted mean body mass index (30.08 vs. 28.07 kg/m^2^) and a higher prevalence of obesity (43.3% vs. 31.7%).

**Table 1:** Weighted characteristics of U.S. adults by treated GERD condition record status, MEPS 2018–2023.

Characteristic	Statistic	No treated GERD record	Treated GERD record	*n* (no record)	*n* (record)	Missing (no record)	Missing (record)
Age, years	Mean (SE)	47.13 (0.16)	61.63 (0.32)	1,09,237	8,725	0	0
Total healthcare expenditure, USD	Mean (SE)	7,825.16 (113.00)	19,298.94 (464.91)	1,09,237	8,725	0	0
Out-of-pocket expenditure, USD	Mean (SE)	1,082.80 (22.91)	2,014.80 (82.64)	1,09,237	8,725	0	0
Body mass index, kg/m^2^	Mean (SE)	28.07 (0.07)	30.08 (0.16)	39,080	3,573	70,157	5,152
Fair or poor physical health	wt% (=1)	11.4%	25.0%	1,09,237	8,725	0	0
Fair or poor mental health	wt% (=1)	9.2%	15.5%	1,09,237	8,725	0	0
High-cost status (top expenditure decile)	wt% (=1)	8.9%	25.7%	1,09,237	8,725	0	0
Obesity (BMI ≥ 30 kg/m^2^)	wt% (=1)	32.6%	44.1%	39,080	3,573	70,157	5,152
Any emergency department use	wt% (=1)	13.0%	27.2%	1,09,237	8,725	0	0
Any inpatient stay	wt% (=1)	6.5%	15.8%	1,09,237	8,725	0	0
Sex: Male	wt%	49.0%	42.2%				
Sex: Female	wt%	51.0%	57.8%				
Race/ethnicity: Hispanic	wt%	17.7%	7.5%				
Race/ethnicity: Non-Hispanic White	wt%	60.5%	77.6%				
Race/ethnicity: Non-Hispanic Black	wt%	12.2%	9.5%				
Race/ethnicity: Non-Hispanic Asian	wt%	6.6%	2.4%				
Race/ethnicity: Non-Hispanic other or multiple	wt%	3.0%	3.0%				
Poverty category: Poor/negative	wt%	10.2%	11.1%				
Poverty category: Near poor	wt%	3.5%	4.2%				
Poverty category: Low income	wt%	11.6%	12.5%				
Poverty category: Middle income	wt%	28.6%	28.0%				
Poverty category: High income	wt%	46.1%	44.2%				
Insurance: Any private	wt%	68.5%	60.1%				
Insurance: Public only	wt%	23.6%	38.9%				
Insurance: Uninsured	wt%	7.9%	1.0%				
Region: Northeast	wt%	17.2%	19.3%				
Region: Midwest	wt%	20.4%	23.9%				
Region: South	wt%	38.1%	41.1%				
Region: West	wt%	24.3%	15.6%				
Diabetes: No	wt%	89.6%	76.7%				
Diabetes: Yes	wt%	10.4%	23.3%				
Hypertension: No	wt%	69.8%	37.2%				
Hypertension: Yes	wt%	30.2%	62.8%				
Heart disease: No	wt%	95.8%	87.1%				
Heart disease: Yes	wt%	4.2%	12.9%				
Stroke: No	wt%	96.6%	90.1%				
Stroke: Yes	wt%	3.4%	9.9%				
Asthma: No	wt%	86.5%	79.1%				
Asthma: Yes	wt%	13.5%	20.9%				
Arthritis: No	wt%	77.7%	42.8%				
Arthritis: Yes	wt%	22.3%	57.2%				
Cancer: No	wt%	89.7%	77.0%				
Cancer: Yes	wt%	10.3%	23.0%				
BMI category: Underweight	wt%	2.9%	2.3%				
BMI category: Normal weight	wt%	32.8%	20.6%				
BMI category: Overweight	wt%	32.6%	33.9%				
BMI category: Obese	wt%	31.7%	43.3%				

Values are survey-weighted percentages or survey-weighted means with standard errors, accompanied by unweighted person-year counts and the number of observations with missing data for each variable. A treated GERD record was defined by an ICD-10-CM K21 condition record linked to a medical event or prescribed-medicine purchase in the survey year. The analytic sample comprises 117,962 adult person-years (62,494 unique persons), of which 8,725 person-years (5,292 unique persons) had a treated GERD record. Body mass index and obesity are reported only for the subsample with a recorded value (39,080 person-years without and 3,573 with a treated GERD record). High-cost status denotes annual total expenditure at or above the pooled survey-weighted 90th percentile ($20,773 in 2023 USD). GERD, gastroesophageal reflux disease; MEPS, medical expenditure panel survey; SE, standard error.

Unadjusted annual total healthcare expenditure averaged $19,298.94 (SE $464.91) among adults with a treated GERD record and $7,825.16 (SE $113.00) among adults without one, and unadjusted out-of-pocket expenditure averaged $2,014.80 (SE $82.64) and $1,082.80 (SE $22.91), respectively. High-cost healthcare status was more common among adults with GERD than among adults without GERD, at 25.7% vs. 8.9%, respectively, as were any emergency department use (27.2% vs. 13.0%), any inpatient stay (15.8% vs. 6.5%), fair or poor perceived physical health (25.0% vs. 11.4%) and fair or poor perceived mental health (15.5% vs. 9.2%). The distribution of total expenditure by GERD status is shown in Supplementary Figure S2.

### Healthcare expenditure

In the fully adjusted quasi-Poisson model, a treated GERD record was associated with an adjusted ratio of mean annual total expenditure of 1.388 (95% CI: 1.318 to 1.462; FDR-corrected *P* < 0.0001), corresponding to 38.8% higher adjusted mean expenditure. The companion linear model gave an adjusted mean difference of $5,481 (95% CI: $4,596 to $6,367; FDR-corrected *P* < 0.0001) in 2023 dollars (Table 2 and Figure 3). Staged models showed the expected attenuation with increasing adjustment: the difference was $8,432 (95% CI: $7,503 to $9,361) in Model 1 and $7,884 (95% CI: $6,965 to $8,802) in Model 2. For annual out-of-pocket expenditure, the adjusted ratio of means was 1.221 (95% CI: 1.126 to 1.324) and the adjusted mean difference was $388 (95% CI: $233 to $542), both with FDR-corrected *P* < 0.0001.

**Table 2:** Association of a treated GERD condition record with annual healthcare expenditure.

Outcome	Model	Measure	Estimate	% change	95% CI	*P* value	FDR *P*	*N*
Total expenditure	Full	Ratio	1.388	38.8	1.318 to 1.462	<0.0001	<0.0001	1,17,134
Out-of-pocket expenditure	Full	Ratio	1.221	22.1	1.126 to 1.324	<0.0001	<0.0001	1,17,134
Total expenditure	Model1	Diff	8,432		7,503 to 9,361	<0.0001		1,17,962
Total expenditure	Model2	Diff	7,884		6,965 to 8,802	<0.0001		1,17,962
Total expenditure	Full	Diff	5,481		4,596 to 6,367	<0.0001	<0.0001	1,17,134
Out-of-pocket expenditure	Full	Diff	388		233 to 542	<0.0001	<0.0001	1,17,134

Expenditures are expressed in 2023 U.S. dollars. Ratios are adjusted ratios of arithmetic mean expenditure from survey-weighted quasi-Poisson regression with a log link; differences are adjusted differences in arithmetic mean expenditure from survey-weighted linear regression. Model 1 adjusted for age, sex and survey year. Model 2 additionally adjusted for race/ethnicity, poverty category, insurance coverage and region. The full model additionally adjusted for comorbid conditions. Perceived physical and mental health were not included in the main models because they are considered potential mediators; the corresponding sensitivity analysis is reported in Supplementary Table S2. FDR *P* denotes the Benjamini–Hochberg false-discovery-rate corrected *P* value. *N* denotes the unweighted analytic sample in person-years. CI, confidence interval; FDR, false-discovery rate; GERD, gastroesophageal reflux disease.

**Figure 2:**
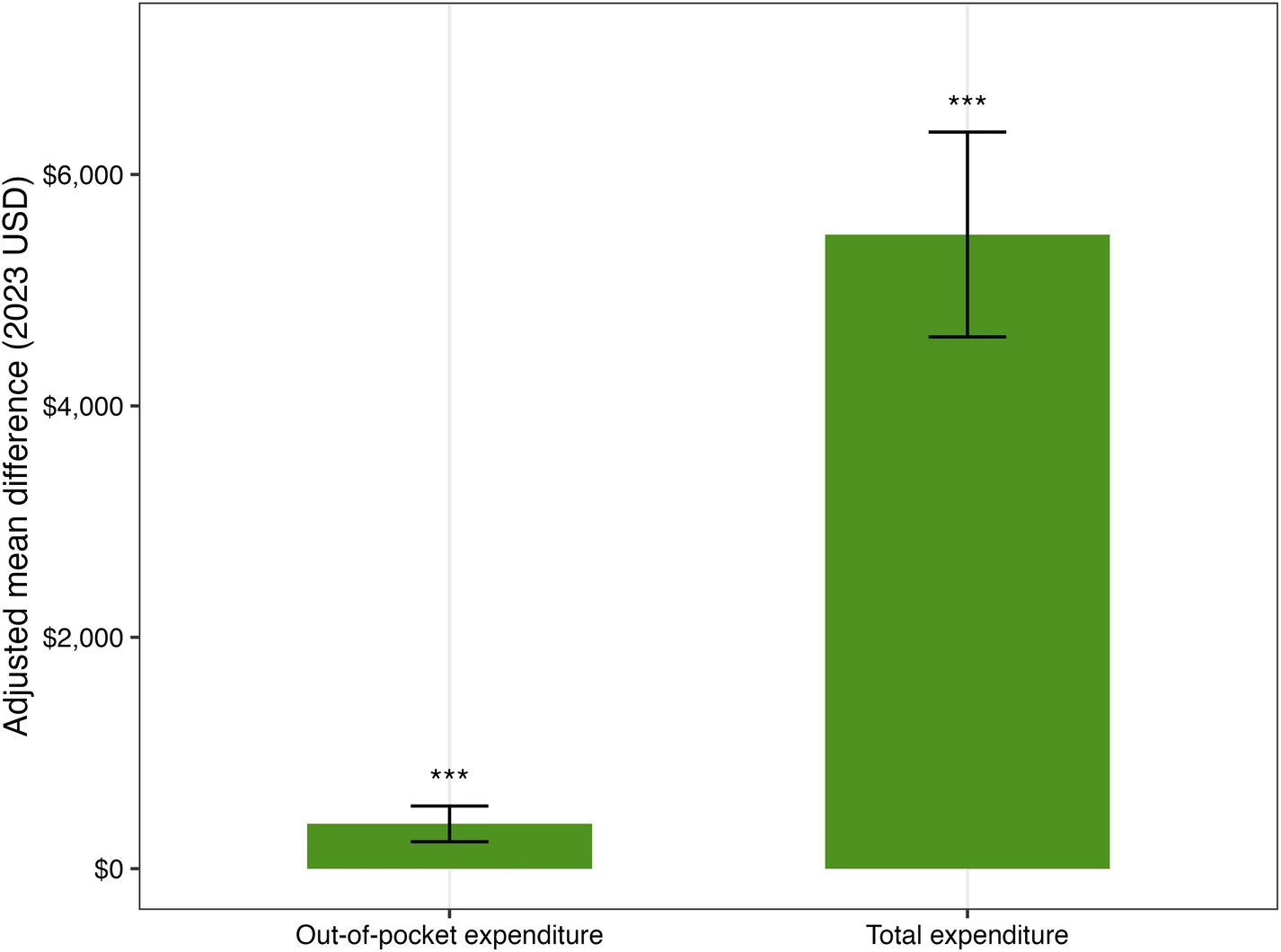
Adjusted associations of a treated GERD condition record with high-cost status and healthcare utilization. Points represent fully adjusted odds ratios and horizontal bars represent 95% confidence intervals, plotted on a logarithmic scale. Models were adjusted for age, sex, survey year, race/ethnicity, poverty category, insurance coverage, region and comorbid conditions. Any prescription medicine purchase is deliberately omitted from this figure because a filled reflux prescription can itself generate the condition record; the corresponding descriptive estimate appears in Supplementary Table S2. The vertical dashed line indicates an odds ratio of 1.0. CI, confidence interval; GERD, gastroesophageal reflux disease; OR, odds ratio. ***P < 0.001.

**Figure 3:**
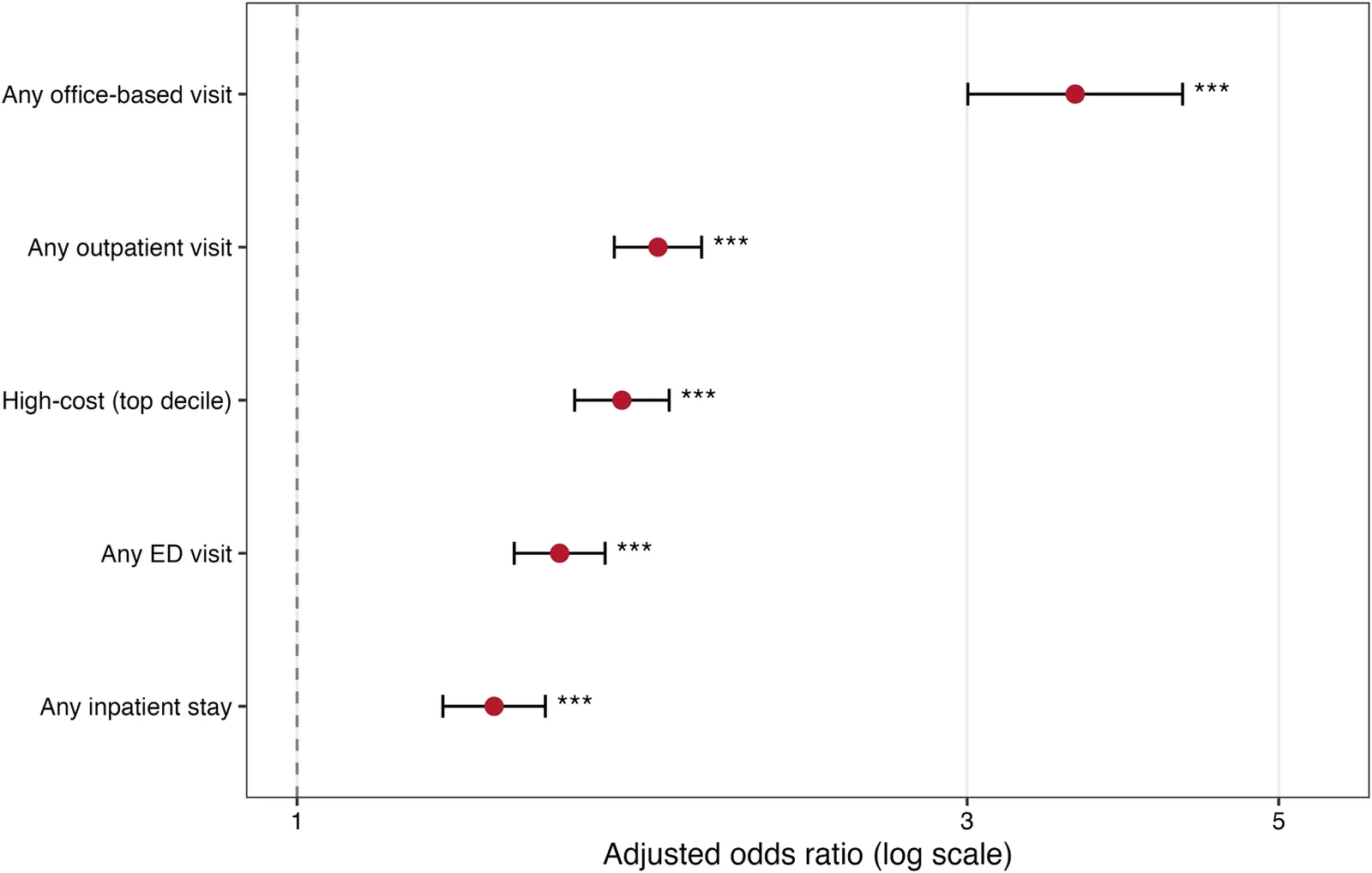
Adjusted differences in annual healthcare expenditure associated with a treated GERD condition record. Bars represent adjusted differences in arithmetic mean annual all-cause total expenditure and out-of-pocket expenditure in 2023 U.S. dollars, from fully adjusted survey-weighted linear regression. Error bars represent 95% confidence intervals. Models were adjusted for age, sex, survey year, race/ethnicity, poverty category, insurance coverage, region and comorbid conditions. Expenditures are all-cause person-level annual expenditures and are not GERD-attributable costs. CI, confidence interval; GERD, gastroesophageal reflux disease; USD, U.S. dollars. ***P < 0.001.

Sensitivity analyses were consistent (Supplementary Table S2 and Supplementary Figure S3). Additional adjustment for perceived physical and mental health, the specification used in the previous version of this analysis, attenuated the adjusted mean difference to $5,055 (95% CI: $4,181 to $5,929), consistent with partial mediation of the association through perceived health. A two-part model yielded an average marginal difference of $4,922. An ordinary least squares model of log-transformed expenditure restricted to the 100,841 person-years with positive expenditure gave a coefficient of 0.569 (95% CI: 0.52 to 0.62); this quantity is a relative difference on the geometric-mean scale and is not directly comparable with the adjusted difference in arithmetic means. Within the 42,565 person-years with recorded body mass index, additional adjustment for body mass index gave an adjusted ratio of mean total expenditure of 1.283 (95% CI: 1.19 to 1.39), indicating that adiposity does not account for the association.

### High-cost status and healthcare utilization

The pooled weighted 90th percentile of annual total expenditure, used to define high-cost status, was $20,773 in 2023 dollars; year-specific thresholds ranged from $19,911 to $21,448 and are reported in Supplementary Table S3. In fully adjusted models, a treated GERD record was associated with higher odds of high-cost status (OR: 1.70; 95% CI: 1.58 to 1.84), emergency department use (OR: 1.54; 95% CI: 1.43 to 1.66), inpatient stay (OR: 1.38; 95% CI: 1.27 to 1.50), office-based visits (OR: 3.58; 95% CI: 3.00 to 4.27) and outpatient visits (OR: 1.81; 95% CI: 1.68 to 1.94), all with FDR-corrected *P* < 0.0001 (Table 3 and Figure 2). Staged models for high-cost status gave odds ratios of 2.34 (95% CI: 2.16 to 2.53) in Model 1 and 2.15 (95% CI: 1.99 to 2.33) in Model 2. Within the subsample with recorded body mass index, the fully adjusted odds ratio for high-cost status was 1.66 (95% CI: 1.46 to 1.89).

**Table 3:** Association of a treated GERD condition record with high-cost status and healthcare utilization.

Outcome	Model	OR	95% CI	*P* value	FDR *P*	*N*
High-cost (top decile)	Model1	2.34	2.16 to 2.53	<0.0001		1,17,962
High-cost (top decile)	Model2	2.15	1.99 to 2.33	<0.0001		1,17,962
High-cost (top decile)	Full	1.7	1.58 to 1.84	<0.0001	<0.0001	1,17,134
Any ED visit	Full	1.54	1.43 to 1.66	<0.0001	<0.0001	1,17,134
Any inpatient stay	Full	1.38	1.27 to 1.50	<0.0001	<0.0001	1,17,134
Any office-based visit	Full	3.58	3.00 to 4.27	<0.0001	<0.0001	1,17,134
Any outpatient visit	Full	1.81	1.68 to 1.94	<0.0001	<0.0001	1,17,134

Estimates are adjusted odds ratios from survey-weighted logistic regression. High-cost status denotes annual total expenditure at or above the pooled survey-weighted 90th percentile ($20,773 in 2023 USD). Model 1, Model 2 and the full model are specified as in Table 2. Any prescription medicine purchase is deliberately excluded from this table and is reported as a descriptive estimate in Supplementary Table S2, because a filled reflux prescription can itself generate the condition record. FDR *P* denotes the Benjamini–Hochberg false-discovery-rate corrected *P* value. *N* denotes the unweighted analytic sample in person-years. CI, confidence interval; FDR, false-discovery rate; GERD, gastroesophageal reflux disease; OR, odds ratio.

Any prescription medicine purchase is reported for completeness only, with a descriptive odds ratio of 61.73 (95% CI: 44.83 to 85.01) in Supplementary Table S2. This estimate is not an inferential result and is not displayed in Figure 2. Because a K21 condition record can be generated by a single filled reflux prescription, the relationship between the exposure and prescription medicine use is largely definitional rather than clinical, and the magnitude of the odds ratio reflects that structural dependence.

### Patient-reported burden

Patient-reported burden was consistently worse among adults with GERD (Supplementary Table S1 and Supplementary Figure S1). In fully adjusted models, a treated GERD record was associated with higher odds of fair or poor physical health (OR: 1.38; 95% CI: 1.27 to 1.50; *n* = 117,134), fair or poor mental health (OR: 1.27; 95% CI: 1.16 to 1.40; *n* = 117,134), activity limitation (OR: 1.65; 95% CI: 1.50 to 1.83; *n* = 106,709), serious psychological distress by K6 (OR: 1.45; 95% CI: 1.24 to 1.69; *n* = 84,411), depressive symptoms by PHQ-2 (OR: 1.45; 95% CI: 1.29 to 1.62; *n* = 85,177), moderate or greater pain interference (OR: 1.54; 95% CI: 1.41 to 1.69; *n* = 85,158) and low energy (OR: 1.46; 95% CI: 1.35 to 1.57; *n* = 85,910), all with FDR-corrected *P* < 0.0001.

Continuous patient-reported scores were directionally consistent. Adjusted differences were 0.635 (95% CI: 0.463 to 0.807) for the K6 score, 0.196 (95% CI: 0.145 to 0.247) for the PHQ-2 score, 0.241 (95% CI: 0.198 to 0.284) for the pain interference score and 0.241 (95% CI: 0.195 to 0.287) for the energy score, each in the direction of greater burden. The social limitation item score also differed (adjusted difference −0.170; 95% CI: −0.210 to −0.129); on this item higher scores denote less interference of health with social activities, so the negative coefficient indicates greater reported interference among adults with a treated GERD record. All continuous patient-reported outcomes, including the social limitation item, reached an FDR-corrected *P* below 0.0001.

### Joint burden of a treated GERD record and depressive symptoms

**Figure 4:**
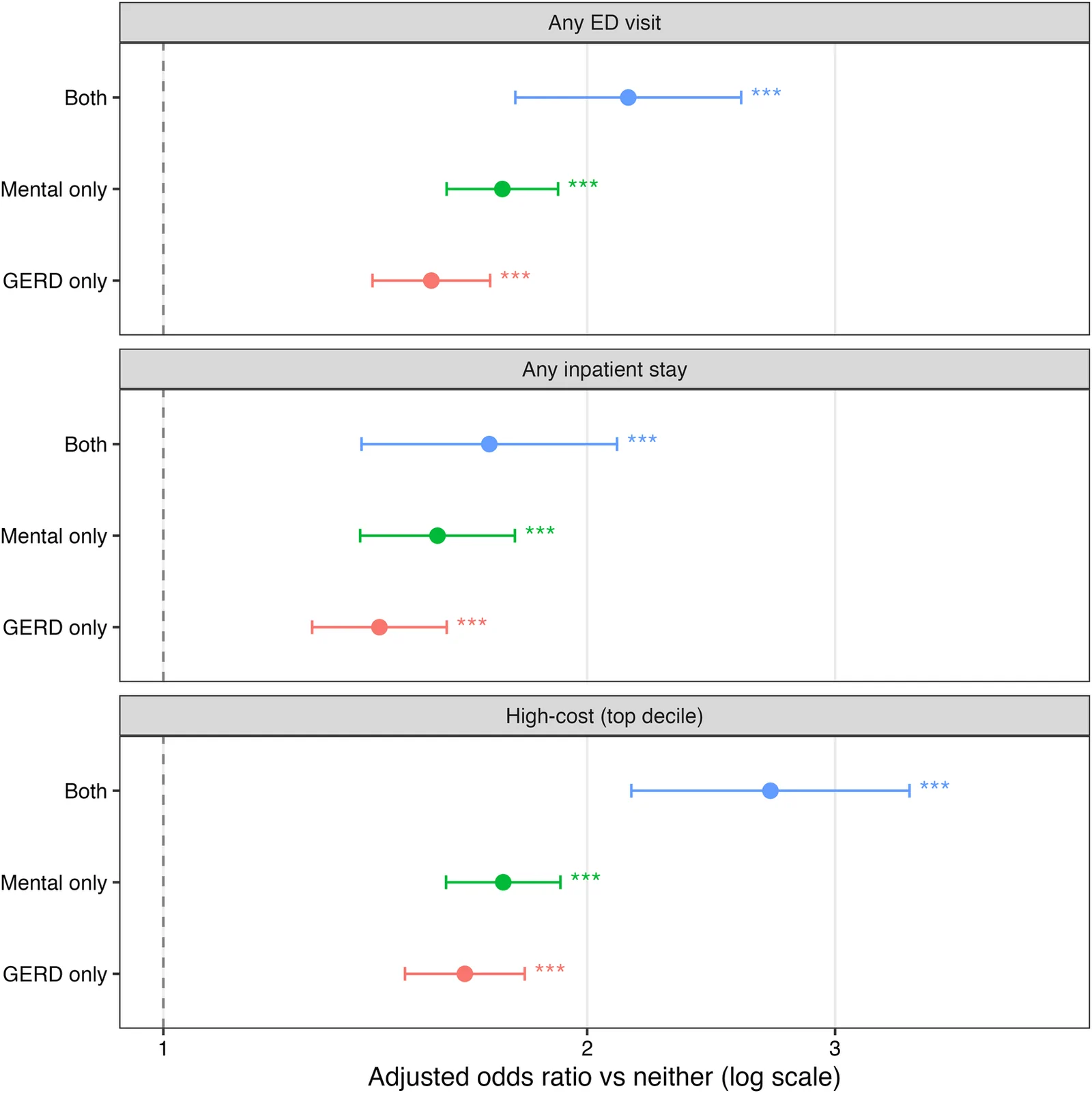
Joint associations of a treated GERD condition record and depressive symptoms with healthcare outcomes. Points represent adjusted odds ratios and horizontal bars represent 95% confidence intervals, plotted on a logarithmic scale, for high-cost status, emergency department use and inpatient stay according to joint exposure category. The reference group is adults with neither a treated GERD record nor depressive symptoms, the latter defined by a PHQ-2 score of 3 or higher. Models used the same covariate set as the fully adjusted main models. No interaction term remained statistically significant after false-discovery-rate correction, so the pattern shown should not be interpreted as evidence of synergy. The vertical dashed line indicates an odds ratio of 1.0. CI, confidence interval; GERD, gastroesophageal reflux disease; OR, odds ratio; PHQ-2, Patient Health Questionnaire-2. ***P < 0.001.

In the primary joint analysis, in which mental burden was defined by PHQ-2 depressive symptoms alone (*n* = 85,177), all three exposed groups had higher burden than adults with neither condition (Table 4 and Figure 4). Odds of high-cost status were 1.64 (95% CI: 1.48 to 1.81) for a treated GERD record alone, 1.74 (95% CI: 1.59 to 1.91) for depressive symptoms alone and 2.70 (95% CI: 2.15 to 3.39) for both. The corresponding odds ratios were 1.55 (95% CI: 1.41 to 1.71), 1.74 (95% CI: 1.59 to 1.91) and 2.14 (95% CI: 1.78 to 2.57) for emergency department use, and 1.42 (95% CI: 1.28 to 1.59), 1.57 (95% CI: 1.38 to 1.78) and 1.70 (95% CI: 1.38 to 2.10) for inpatient stay. Adjusted differences in annual total expenditure were $4,035 (95% CI: $3,032 to $5,039), $3,908 (95% CI: $2,961 to $4,854) and $11,274 (95% CI: $8,059 to $14,489), respectively. All estimates had FDR-corrected *P* values below 0.0001.

**Table 4:** Joint association of a treated GERD condition record and depressive symptoms with healthcare outcomes.

Outcome	Group	Measure	Estimate	95% CI	FDR *P*
High-cost (top decile)	GERD only	OR	1.64	1.48 to 1.81	<0.0001
High-cost (top decile)	Mental burden only	OR	1.74	1.59 to 1.91	<0.0001
High-cost (top decile)	Both	OR	2.70	2.15 to 3.39	<0.0001
Any ED visit	GERD only	OR	1.55	1.41 to 1.71	<0.0001
Any ED visit	Mental burden only	OR	1.74	1.59 to 1.91	<0.0001
Any ED visit	Both	OR	2.14	1.78 to 2.57	<0.0001
Any inpatient stay	GERD only	OR	1.42	1.28 to 1.59	<0.0001
Any inpatient stay	Mental burden only	OR	1.57	1.38 to 1.78	<0.0001
Any inpatient stay	Both	OR	1.70	1.38 to 2.10	<0.0001
Total expenditure	GERD only	Diff	4,035	3,032 to 5,039	<0.0001
Total expenditure	Mental burden only	Diff	3,908	2,961 to 4,854	<0.0001
Total expenditure	Both	Diff	11,274	8,059 to 14,489	<0.0001

The reference group is adults with neither a treated GERD record nor depressive symptoms. Mental burden is defined in this primary analysis solely by a PHQ-2 score of 3 or higher, among the 85,177 person-years with a valid PHQ-2 score; the corresponding analysis using K6 serious psychological distress is reported in Supplementary Table S5. Odds ratios are from survey-weighted logistic regression and expenditure differences are adjusted differences in arithmetic mean annual total expenditure in 2023 U.S. dollars. Models used the same covariate set as the fully adjusted models in Tables 2, 3, namely age, sex, survey year, race/ethnicity, poverty category, insurance coverage, region and comorbid conditions. FDR *P* denotes the Benjamini–Hochberg false-discovery-rate corrected *P* value. CI, confidence interval; FDR, false-discovery rate; GERD, gastroesophageal reflux disease; OR, odds ratio; PHQ-2, patient health questionnaire-2.

Formal interaction testing did not support synergy (Table 5). The multiplicative interaction term was not significant for high-cost status, the primary utilization outcome (*P* = 0.66). Nominally significant multiplicative interaction terms were observed for emergency department use (*P* = 0.025) and inpatient stay (*P* = 0.045), but both were in the submultiplicative direction, indicating a joint association smaller than the product of the two individual associations, and neither remained significant after Benjamini–Hochberg correction across the three interaction tests (corrected *P* = 0.068 for both). The pattern is therefore one of two conditions each independently associated with higher burden, combining in an approximately additive fashion, rather than one of synergistic effect. The sensitivity analysis defining mental burden by K6 serious psychological distress alone (*n* = 84,411) produced closely similar estimates, with odds of high-cost status of 1.64, 1.78 and 2.73 for the three exposed groups and an adjusted expenditure difference of $10,819 (95% CI: $7,203 to $14,435) in the joint group (Supplementary Table S5).

**Table 5:** Tests of interaction between a treated GERD condition record and depressive symptoms.

Outcome	Scale	Interaction *P*
High-cost (top decile)	Multiplicative(logit)	0.658
Any ED visit	Multiplicative(logit)	0.0248
Any inpatient stay	Multiplicative(logit)	0.045

*P* values are from Wald tests of the product term on the multiplicative (logit) scale in the fully adjusted joint models described in Table 4. Corrected *P* values were obtained by applying the Benjamini–Hochberg procedure across the three tests and were 0.658, 0.068 and 0.068 for high-cost status, emergency department use and inpatient stay, respectively. The interaction terms for emergency department use and inpatient stay were in the submultiplicative direction. GERD, gastroesophageal reflux disease.

## Discussion

In this pooled nationally representative analysis of U.S. adults from 2018 to 2023, adults with a treated, event-linked K21 reflux condition record had higher annual all-cause healthcare expenditure, greater odds of high-cost healthcare status, more frequent emergency department, inpatient, office-based and outpatient use, and worse patient-reported physical, mental, pain, energy and functional outcomes. These associations persisted after adjustment for demographic, socioeconomic, insurance, regional and comorbidity factors, and were robust to alternative expenditure model specifications and to adjustment for body mass index. Adults who also reported depressive symptoms carried the highest absolute burden, although formal interaction testing gave no evidence of synergy.

The weighted proportion of adults carrying a treated K21 record, 6.4% to 6.7%, is much lower than symptom-based prevalence estimates for the United States, and this gap is informative rather than anomalous. Symptom surveys enumerate everyone who experiences troublesome heartburn or regurgitation, a group comprising roughly one fifth to one third of U.S. adults (6, 7). MEPS condition records, by contrast, capture only reflux disease that generated a medical event or a prescribed-medicine purchase within the calendar year (14). The intervening filters are substantial: many adults with reflux symptoms self-manage with over-the-counter acid suppression, do not consult a clinician, or consult without the condition being coded. Our estimate should therefore be read as the proportion of adults receiving recorded care for reflux disease in a given year, and every finding in this study applies to that population rather than to all adults with GERD.

The same feature of the data constrains how the utilization findings should be read. Because the exposure is generated by healthcare contact, associations between the exposure and outcomes that also reflect healthcare contact are partly structural rather than clinical. This dependence is most extreme for prescription medicine use, where a single filled reflux prescription can create the condition record; we therefore removed that outcome from all inferential analyses and report it descriptively only. It is also relevant, although far less extreme, for office-based visits, since reflux disease is typically diagnosed and coded in ambulatory care; the odds ratio of 3.58 for office-based visits should be interpreted with that caveat in mind. Outcomes that are less tightly bound to the recording mechanism, particularly high-cost status, emergency department use, inpatient stay and the patient-reported measures, are correspondingly less vulnerable to this concern, and it is on these that we base the substantive interpretation. Even for these outcomes, however, adults who interact more frequently with the healthcare system are both more likely to accrue a coded reflux diagnosis and more likely to accrue expenditure, and residual outcome-dependent ascertainment cannot be excluded.

The expenditure findings should be interpreted as excess all-cause spending among adults with a treated GERD record rather than as GERD-attributable costs. After full adjustment, mean annual total expenditure was 38.8% higher, an absolute difference of $5,481, and out-of-pocket expenditure was $388 higher. The two estimates answer slightly different questions: the ratio describes proportional difference in adjusted mean spending, while the dollar difference describes the absolute burden borne by the health system, and both are differences in arithmetic means rather than medians. Because perceived physical and mental health plausibly lie on the causal pathway, they are not adjusted for in the main model; adding them attenuates the difference to $5,055, and the two-part model and the body mass index sensitivity analysis point in the same direction. MEPS captures person-level annual expenditure across all conditions, so these figures describe the overall expenditure profile of adults with a treated reflux record and are consistent with prior U.S. digestive disease burden work showing that upper gastrointestinal disorders contribute substantially to healthcare use and spending (11). They do not establish that reflux disease itself caused the full difference, and they are not condition-specific costs; estimating GERD-attributable expenditure would require linking condition records to event-level spending through the MEPS condition-event link files, which we identify as a priority for further work.

A key contribution of this study is the integration of expenditure, utilization, and patient-reported burden within the same nationally representative framework. Prior studies have shown that GERD can impair health-related quality of life, sleep, and daytime functioning (25–27). Our findings extend this patient-centered perspective by showing consistent associations of GERD with fair or poor physical health, fair or poor mental health, serious psychological distress, depressive symptoms, pain interference, low energy, and activity limitation. These results support the view that GERD burden should not be evaluated only through acid-suppression needs, endoscopic findings, or healthcare spending. For many patients, the clinical impact of GERD may be reflected in daily symptoms, sleep disturbance, discomfort, reduced energy, and functional limitation. Because the patient-reported instruments are administered only to respondents completing the self-administered questionnaires, these estimates rest on smaller analytic samples than the expenditure and utilization estimates, and the corresponding sample sizes are reported alongside each estimate.

The joint analysis of a treated GERD record and depressive symptoms adds a clinically relevant perspective, but it must be described carefully. Adults with both had the highest absolute burden, with an adjusted expenditure difference of $11,274 and odds of high-cost status of 2.70. Formal testing, however, showed no multiplicative interaction for high-cost status, and the nominally significant interaction terms for emergency department and inpatient use were submultiplicative and did not survive correction for multiple testing. The data are therefore consistent with two conditions that are each independently associated with higher burden and that combine approximately additively, and we explicitly do not claim synergy. This pattern still aligns with a substantial literature associating GERD with anxiety, depression, psychological stress, sleep disturbance and psychiatric comorbidity (28–30), and with evidence that psychological processes contribute importantly to symptom severity in refractory heartburn or regurgitation (3). The clinical implication we draw is correspondingly modest and hypothesis-generating: co-occurring depressive symptoms may help flag patients with treated reflux disease who carry unusually high overall burden and who might benefit from broader assessment. Whether integrated psychological assessment improves outcomes in this group cannot be determined from a cross-sectional design and requires prospective evaluation.

This study has several strengths. First, it used six recent years of nationally representative MEPS data, allowing contemporary estimation of GERD-associated burden among U.S. adults. Second, it evaluated multiple burden domains rather than focusing only on prevalence, expenditure, or medication use. Third, the analyses incorporated the complex survey design, the AHRQ pooled linkage variance structure required when pooling across the 2019 design boundary, pooled survey weights, inflation-adjusted expenditures, staged covariate adjustment, and sensitivity analyses. Fourth, expenditure was modelled on its original scale with a mean-preserving generalized linear model and cross-checked against two-part and log-transformed specifications. Fifth, the exposure definition, its dependence on healthcare contact, and the resulting limits on interpretation are stated explicitly rather than left implicit.

Several limitations should be acknowledged. The most important is the exposure definition itself. A treated GERD record is generated only through healthcare contact, so the study population is adults receiving recorded care for reflux disease, the associations with contact-dependent outcomes are partly structural, and the findings do not generalize to all adults with GERD. MEPS provides no reflux symptom instrument, so an outcome-independent case definition was not available. GERD was defined using public-use three-character ICD-10-CM K21 condition codes, so K21.0 and K21.9 could not be distinguished and GERD severity, symptom frequency, treatment response, and reflux phenotype could not be determined. The data are cross-sectional within each survey year, so temporal ordering and causality cannot be established. Expenditures were all-cause person-level annual expenditures rather than GERD-specific costs. Residual confounding may remain because MEPS does not fully capture all factors relevant to GERD burden, such as diet, sleep quality, symptom severity, medication adherence, over-the-counter medication use, healthcare-seeking behavior, and detailed treatment patterns; body mass index was recorded for only part of the sample and could be addressed only in a subsample sensitivity analysis. Patient-reported measures were available only for respondents completing the self-administered questionnaires, and although missingness was somewhat lower among adults with a treated GERD record than among those without (Supplementary Table S4), differential non-response cannot be excluded. Finally, because MEPS panels span two calendar years, some individuals contribute two person-year observations; the pooled linkage variance structure addresses the resulting design complexity, but the analysis treats person-years rather than persons as the unit of observation.

## Conclusions

Among U.S. adults from 2018 to 2023, a treated, event-linked reflux condition record identified a population with substantially higher all-cause healthcare expenditure, greater healthcare utilization and worse patient-reported physical, mental, pain, energy and functional outcomes. Adults who additionally reported depressive symptoms carried the highest absolute burden, although the two conditions appeared to combine approximately additively rather than synergistically. Because case identification in national survey condition files requires healthcare contact, these findings characterize the burden borne by adults receiving care for reflux disease rather than the causal effect of GERD, and they support assessing that burden multidimensionally across economic, utilization, functional and psychological domains.

## Data Availability

The datasets presented in this study can be found in online repositories. The names of the repository/repositories and accession number(s) can be found in the article/Supplementary Material.
