# Supplementary material for: Healthcare burden of treated gastroesophageal reflux disease among U.S. adults, 2018–2023: a pooled cross-sectional analysis of the medical expenditure panel survey
Source: Front Med (Lausanne). 2026 Sep 4;13:1933050. doi: 10.3389/fmed.2026.1933050 (PMC13585660; doi:10.3389/fmed.2026.1933050)
Supplement: Supplementary file 1 [file Supplementaryfile1.docx]

# Supplementary table titles and notes

## Supplementary Table S1. Association of a treated GERD condition record with patient-reported burden outcomes.

| Outcome | Measure | Estimate | 95% CI | P value | FDR P | N |
| --- | --- | --- | --- | --- | --- | --- |
| Fair/poor physical health | OR | 1.38 | 1.27 to 1.50 | <0.0001 | <0.0001 | 117134 |
| Fair/poor mental health | OR | 1.27 | 1.16 to 1.40 | <0.0001 | <0.0001 | 117134 |
| Activity limitation | OR | 1.65 | 1.50 to 1.83 | <0.0001 | <0.0001 | 106709 |
| Serious distress (K6>=13) | OR | 1.45 | 1.24 to 1.69 | <0.0001 | <0.0001 | 84411 |
| Depressive symptoms (PHQ-2>=3) | OR | 1.45 | 1.29 to 1.62 | <0.0001 | <0.0001 | 85177 |
| Moderate+ pain interference | OR | 1.54 | 1.41 to 1.69 | <0.0001 | <0.0001 | 85158 |
| Low energy | OR | 1.46 | 1.35 to 1.57 | <0.0001 | <0.0001 | 85910 |
| K6 score | beta | 0.635 | 0.463 to 0.807 | <0.0001 | <0.0001 | 84411 |
| PHQ-2 score | beta | 0.196 | 0.145 to 0.247 | <0.0001 | <0.0001 | 85177 |
| Pain interference score | beta | 0.241 | 0.198 to 0.284 | <0.0001 | <0.0001 | 85158 |
| Energy score | beta | 0.241 | 0.195 to 0.287 | <0.0001 | <0.0001 | 85910 |
| Social limitation score | beta | -0.170 | -0.210 to -0.129 | <0.0001 | <0.0001 | 85992 |

Odds ratios are from survey-weighted logistic regression for binary outcomes and beta coefficients are adjusted mean differences from survey-weighted linear regression for continuous scores. Models adjusted for age, sex, survey year, race/ethnicity, poverty category, insurance coverage, region and comorbid conditions. Analytic sample sizes differ because the K6, PHQ-2 and VR-12 items are administered only through the self-administered questionnaires. For the social limitation item, higher scores denote less interference of health with social activities, so a negative coefficient indicates greater reported interference. FDR P denotes the Benjamini–Hochberg false-discovery-rate corrected P value. CI, confidence interval; GERD, gastroesophageal reflux disease; K6, Kessler Psychological Distress Scale; OR, odds ratio; PHQ-2, Patient Health Questionnaire-2.

## Supplementary Table S2. Sensitivity and descriptive analyses.

| Outcome | Model | Measure | Estimate | 95% CI | P value | N |
| --- | --- | --- | --- | --- | --- | --- |
| Total expenditure | Full+PH(sens) | diff | 5054.924 | 4180.56 to 5929.29 | <0.0001 | 117134 |
| Total expenditure (log scale) | log-OLS(sens) | log-beta | 0.569 | 0.52 to 0.62 | <0.0001 | 100841 |
| Total expenditure | two-part(sens) | diff(two-part AMD) | 4921.741 |  |  | 117134 |
| Total expenditure | Full+BMI(sens,SAQ) | ratio | 1.283 | 1.19 to 1.39 | <0.0001 | 42565 |
| High-cost (top decile) | Full+BMI(sens,SAQ) | OR | 1.657 | 1.46 to 1.89 | <0.0001 | 42565 |
| Any prescription medicine purchase | DESCRIPTIVE | OR | 61.734 | 44.83 to 85.01 | <0.0001 | 117134 |
| High-cost (top decile) | joint-K6(sens) | OR | 1.638 | 1.49 to 1.80 | <0.0001 | 84411 |
| High-cost (top decile) | joint-K6(sens) | OR | 1.783 | 1.60 to 1.99 | <0.0001 | 84411 |
| High-cost (top decile) | joint-K6(sens) | OR | 2.729 | 2.01 to 3.72 | <0.0001 | 84411 |
| Any ED visit | joint-K6(sens) | OR | 1.53 | 1.39 to 1.69 | <0.0001 | 84411 |
| Any ED visit | joint-K6(sens) | OR | 1.949 | 1.73 to 2.19 | <0.0001 | 84411 |
| Any ED visit | joint-K6(sens) | OR | 2.314 | 1.83 to 2.93 | <0.0001 | 84411 |
| Any inpatient stay | joint-K6(sens) | OR | 1.396 | 1.26 to 1.55 | <0.0001 | 84411 |
| Any inpatient stay | joint-K6(sens) | OR | 1.743 | 1.50 to 2.03 | <0.0001 | 84411 |
| Any inpatient stay | joint-K6(sens) | OR | 1.85 | 1.43 to 2.39 | <0.0001 | 84411 |
| Total expenditure | joint-K6(sens) | diff | 4191.283 | 3185.95 to 5196.62 | <0.0001 | 84411 |
| Total expenditure | joint-K6(sens) | diff | 4037.337 | 3024.58 to 5050.09 | <0.0001 | 84411 |
| Total expenditure | joint-K6(sens) | diff | 10818.672 | 7202.52 to 14434.82 | <0.0001 | 84411 |

Full+PH denotes the fully adjusted model with additional adjustment for perceived physical and mental health. The log-OLS row reports an ordinary least squares model of log-transformed expenditure restricted to person-years with positive expenditure; its coefficient is a relative difference on the geometric-mean scale and is not a percentage difference in arithmetic mean expenditure. The two-part row reports the average marginal difference from a logistic model for any expenditure combined with a log-link generalized linear model for positive expenditure. Full+BMI rows are restricted to the subsample with a recorded body mass index. The prescription medicine row is descriptive only and must not be interpreted as an independent association, because a filled reflux prescription can itself generate the K21 condition record. The joint-K6 rows correspond to Supplementary Table S5. BMI, body mass index; OLS, ordinary least squares; SAQ, Self-Administered Questionnaire.

## Supplementary Table S3. Year-specific weighted proportion of adults with a treated GERD condition record and year-specific high-cost thresholds.

| Year | Recorded GERD % | 95% CI | High-cost threshold (2023 USD) |
| --- | --- | --- | --- |
| 2018 | 6.65 | 6.13 to 7.17 | 20781 |
| 2019 | 6.5 | 6.04 to 6.96 | 21448 |
| 2020 | 6.48 | 5.97 to 6.99 | 19911 |
| 2021 | 6.46 | 6.00 to 6.92 | 21437 |
| 2022 | 6.46 | 5.95 to 6.98 | 20047 |
| 2023 | 6.4 | 5.86 to 6.94 | 21064 |

The recorded GERD percentage is the survey-weighted proportion of U.S. adults with an ICD-10-CM K21 condition record linked to a medical event or prescribed-medicine purchase in that survey year. High-cost thresholds are year-specific survey-weighted 90th percentiles of annual total expenditure expressed in 2023 U.S. dollars; the pooled threshold used in the main analysis was $20,773. CI, confidence interval; GERD, gastroesophageal reflux disease; USD, U.S. dollars.

## Supplementary Table S4. Percentage of missing values for analytic variables by treated GERD condition record status.

| Characteristic | Missing, no record (%) | Missing, record (%) |
| --- | --- | --- |
| Total healthcare expenditure, USD | 0 | 0 |
| Out-of-pocket expenditure, USD | 0 | 0 |
| Fair or poor physical health | 0 | 0 |
| Fair or poor mental health | 0 | 0 |
| High-cost status (top expenditure decile) | 0 | 0 |
| K6 distress score | 28.9206038 | 19.9541547 |
| PHQ-2 score | 28.2907806 | 19.0487106 |
| Serious psychological distress (K6 ≥ 13) | 28.9206038 | 19.9541547 |
| Depressive symptoms (PHQ-2 ≥ 3) | 28.2907806 | 19.0487106 |
| Moderate or greater pain interference | 28.3100049 | 19.0372493 |
| Low energy | 27.6719427 | 18.3954155 |
| Activity limitation | 8.93012441 | 9.53581662 |
| VR-12 physical component summary | 100 | 100 |
| VR-12 mental component summary | 100 | 100 |
| Social limitation | 27.6463103 | 17.7879656 |
| Body mass index, kg/m² | 64.2245759 | 59.0487106 |

Values are the percentage of person-year observations with a missing value for each variable, shown separately for adults without and with a treated GERD condition record. Missingness for the K6, PHQ-2, pain, energy and social limitation items reflects administration of these instruments through the self-administered questionnaires. The VR-12 physical and mental component summary scores were not available in the pooled public-use files for these survey years and were therefore not analysed. GERD, gastroesophageal reflux disease; VR-12, Veterans RAND 12-item Health Survey.

## Supplementary Table S5. Joint association of a treated GERD condition record and K6 serious psychological distress with healthcare outcomes.

| Outcome | Group | Measure | Estimate | 95% CI | P value |
| --- | --- | --- | --- | --- | --- |
| High-cost (top decile) | GERD only | OR | 1.64 | 1.49 to 1.80 | <0.0001 |
| High-cost (top decile) | Mental burden only | OR | 1.78 | 1.60 to 1.99 | <0.0001 |
| High-cost (top decile) | Both | OR | 2.73 | 2.01 to 3.72 | <0.0001 |
| Any ED visit | GERD only | OR | 1.53 | 1.39 to 1.69 | <0.0001 |
| Any ED visit | Mental burden only | OR | 1.95 | 1.73 to 2.19 | <0.0001 |
| Any ED visit | Both | OR | 2.31 | 1.83 to 2.93 | <0.0001 |
| Any inpatient stay | GERD only | OR | 1.40 | 1.26 to 1.55 | <0.0001 |
| Any inpatient stay | Mental burden only | OR | 1.74 | 1.50 to 2.03 | <0.0001 |
| Any inpatient stay | Both | OR | 1.85 | 1.43 to 2.39 | <0.0001 |
| Total expenditure | GERD only | diff | 4191 | 3186 to 5197 | <0.0001 |
| Total expenditure | Mental burden only | diff | 4037 | 3025 to 5050 | <0.0001 |
| Total expenditure | Both | diff | 10819 | 7203 to 14435 | <0.0001 |

Sensitivity analysis corresponding to Table 4, in which mental burden is defined solely by a K6 score of 13 or higher among the 84,411 person-years with a valid K6 score. The reference group is adults with neither a treated GERD record nor serious psychological distress. Covariate adjustment is identical to Table 4. CI, confidence interval; GERD, gastroesophageal reflux disease; K6, Kessler Psychological Distress Scale; OR, odds ratio.

## Supplementary Table S6. Unweighted analytic sample size for every model.

| Block | Outcome | Model | Analytic N (person-years) |
| --- | --- | --- | --- |
| expenditure | Total expenditure | Full | 117134 |
| expenditure | Out-of-pocket expenditure | Full | 117134 |
| expenditure | Total expenditure | Model1 | 117962 |
| expenditure | Total expenditure | Model2 | 117962 |
| expenditure | Total expenditure | Full+PH(sens) | 117134 |
| expenditure | Total expenditure (log scale) | log-OLS(sens) | 100841 |
| expenditure | Total expenditure | two-part(sens) | 117134 |
| expenditure | Total expenditure | Full+BMI(sens,SAQ) | 42565 |
| utilization | High-cost (top decile) | Full+BMI(sens,SAQ) | 42565 |
| utilization | High-cost (top decile) | Model1 | 117962 |
| utilization | High-cost (top decile) | Model2 | 117962 |
| utilization | High-cost (top decile) | Full | 117134 |
| utilization | Any ED visit | Full | 117134 |
| utilization | Any inpatient stay | Full | 117134 |
| utilization | Any office-based visit | Full | 117134 |
| utilization | Any outpatient visit | Full | 117134 |
| descriptive | Any prescription medicine purchase | DESCRIPTIVE | 117134 |
| patient-reported | Fair/poor physical health | Full | 117134 |
| patient-reported | Fair/poor mental health | Full | 117134 |
| patient-reported | Activity limitation | Full | 106709 |
| patient-reported | Serious distress (K6>=13) | Full | 84411 |
| patient-reported | Depressive symptoms (PHQ-2>=3) | Full | 85177 |
| patient-reported | Moderate+ pain interference | Full | 85158 |
| patient-reported | Low energy | Full | 85910 |
| patient-reported | K6 score | Full | 84411 |
| patient-reported | PHQ-2 score | Full | 85177 |
| patient-reported | Pain interference score | Full | 85158 |
| patient-reported | Energy score | Full | 85910 |
| patient-reported | Social limitation score | Full | 85992 |
| joint | High-cost (top decile) | joint-PHQ2 | 85177 |
| joint | High-cost (top decile) | joint-K6(sens) | 84411 |
| joint | Any ED visit | joint-PHQ2 | 85177 |
| joint | Any ED visit | joint-K6(sens) | 84411 |
| joint | Any inpatient stay | joint-PHQ2 | 85177 |
| joint | Any inpatient stay | joint-K6(sens) | 84411 |
| joint | Total expenditure | joint-PHQ2 | 85177 |
| joint | Total expenditure | joint-K6(sens) | 84411 |

Analytic N is the unweighted number of person-year observations contributing to each complete-case model. Differences across outcomes reflect the availability of the self-administered questionnaire instruments and of body mass index. GERD, gastroesophageal reflux disease.

# Supplementary figure legends

## Supplementary Figure S1. Adjusted associations of a treated GERD condition record with patient-reported burden outcomes.

Points represent fully adjusted odds ratios and horizontal bars represent 95% confidence intervals, plotted on a logarithmic scale, for perceived physical and mental health, serious psychological distress, depressive symptoms, pain interference, low energy and activity limitation. Models were adjusted for age, sex, survey year, race/ethnicity, poverty category, insurance coverage, region and comorbid conditions. Analytic sample sizes differ across outcomes and are reported in Supplementary Tables S1 and S6. The vertical dashed line indicates an odds ratio of 1.0. CI, confidence interval; GERD, gastroesophageal reflux disease; K6, Kessler Psychological Distress Scale; OR, odds ratio; PHQ-2, Patient Health Questionnaire-2. ***P < 0.001.

## Supplementary Figure S2. Distribution of annual total healthcare expenditure by treated GERD condition record status.

Violin plots with superimposed box plots show the distribution of log-transformed annual total expenditure, in 2023 U.S. dollars, among adults with and without a treated GERD condition record. The figure illustrates both the strong right skew and the mass of low or zero expenditure that motivated modelling expenditure on its original scale with a log-link generalized linear model. GERD, gastroesophageal reflux disease; USD, U.S. dollars.

## Supplementary Figure S3. Comparison of expenditure model specifications.

Bars represent adjusted differences in arithmetic mean annual total expenditure in 2023 U.S. dollars from the fully adjusted linear model and from the same model with additional adjustment for perceived physical and mental health. Error bars represent 95% confidence intervals. The attenuation observed on adding perceived health is consistent with partial mediation of the association through perceived health status. CI, confidence interval; USD, U.S. dollars.

## Supplementary Figure S4. Analytic sample flow for the pooled MEPS 2018–2023 analysis.

The flow diagram shows construction of the adult analytic sample from the pooled MEPS full-year files. Among 156,464 pooled person-year records, 121,770 were for adults aged 18 years or older, and 117,962 adult person-year observations representing 62,494 unique persons had positive person-level survey weights and valid survey design information and formed the analytic sample. A treated GERD condition record, identified by an ICD-10-CM K21 code linked to a medical event or prescribed-medicine purchase, was present in 8,725 person-year observations representing 5,292 unique persons. GERD, gastroesophageal reflux disease; ICD-10-CM, International Classification of Diseases, Tenth Revision, Clinical Modification; MEPS, Medical Expenditure Panel Survey.
